# Associations of Daily Sleep and Stress With Rumination: An Ecological Momentary Assessment and Actigraphy Study

**DOI:** 10.1093/geroni/igab046.432

**Published:** 2021-12-17

**Authors:** Taylor Vigoureux, Soomi Lee

**Affiliations:** University of South Florida, Tampa, Florida, United States

## Abstract

Unconstructive repetitive thoughts are indicative of rumination about daily experiences. Given that poor sleep is associated with greater reactivity to daily stressors, we examined joint associations of daily sleep and stress with daily rumination. 143 nurses completed 14 days of ecological momentary assessments (EMA; assessments of daily sleep, stress, and rumination) and actigraphy. After controlling for age, sex, education, income, inpatient vs. outpatient nurse, workday, and day vs. night shift, there were significant joint associations of sleep and stress with daily rumination. Daily rumination was lowest when lower (-1SD) stressor severity or stressor frequency were paired with longer (+1SD) EMA or actigraphy sleep duration. Daily rumination was highest when higher stressor frequency was paired with higher actigraphy wake after sleep onset (i.e., poorer sleep quality). Future studies should assess whether rumination about daily experiences is associated with quality of patient care provided by nurses in a hospital setting.

